# Subcutaneous Angiofibroma of the Ankle: A Rare, Undescribed Clinical Entity

**DOI:** 10.7759/cureus.53033

**Published:** 2024-01-27

**Authors:** Sanjay V Deshpande, Vivek H Jadawala, Salahuddin Ahmed, Sachin Goel

**Affiliations:** 1 Department of Orthopaedic Surgery, Jawaharlal Nehru Medical College, Datta Meghe Institute of Medical Sciences, Wardha, IND

**Keywords:** spindle cells, benign tumor, fibrovascular tumor, liposarcoma, soft tissue angiofibroma

## Abstract

Soft tissue angiofibroma is a pathology consisting of a benign fibrous vascularized tumor that mimics low-grade sarcoma. Such tumors frequently arise in the extremities, more commonly in the lower extremities, presenting as a slow-growing, painless swelling. Females are more commonly affected than males. We present the case of a 42-year-old male with a slow-growing, painless mass on the extensor aspect of his left foot. Differential diagnoses considered were soft tissue fibrosarcoma, liposarcoma, and sebaceous cysts. Surgical excision of the tumor was done, and upon histopathology, there were multiple lobules with well-circumscribed, alternating areas of collagenous and myxoid tissues. There was a prominent small vascular network with uniformly arranged spindle cells consisting of pale eosinophil-rich cytoplasm and small ovoid nuclei, fine chromatin, and an indistinct nucleolus. There are not many reported cases of this clinical entity, and every new case reported brings light to the pathology and progression of this tumor. Understanding this pathology is necessary since it mimics many other skin and soft tissue tumors.

## Introduction

Soft tissue angiofibroma was first described in 2012 as a pathology consisting of a benign fibrous vascularized tumor that mimics sarcoma of low grade [1]. Such tumors frequently arise in the extremities, more commonly in the lower extremities, presenting as a slow-growing, painless swelling [2]. Infrequently, it arises in proximity to large joints like the knee and is often subcutaneous, but may be intra-muscular or deep. Females are more commonly affected than males. Angiofibromas are usually well circumscribed, with morphological features resembling angiofibromas of the nasal cavity. Immunohistochemically (IHC), these tumor cells show positive foci for epithelial membrane antigen (EMA); some rare cases may even have atypical cells that stain CD34, smooth muscle actin (SMA), and desmin [1]. We present a rare case of subcutaneous angiofibroma at the extensor aspect of the ankle.

## Case presentation

**Figure 1:**
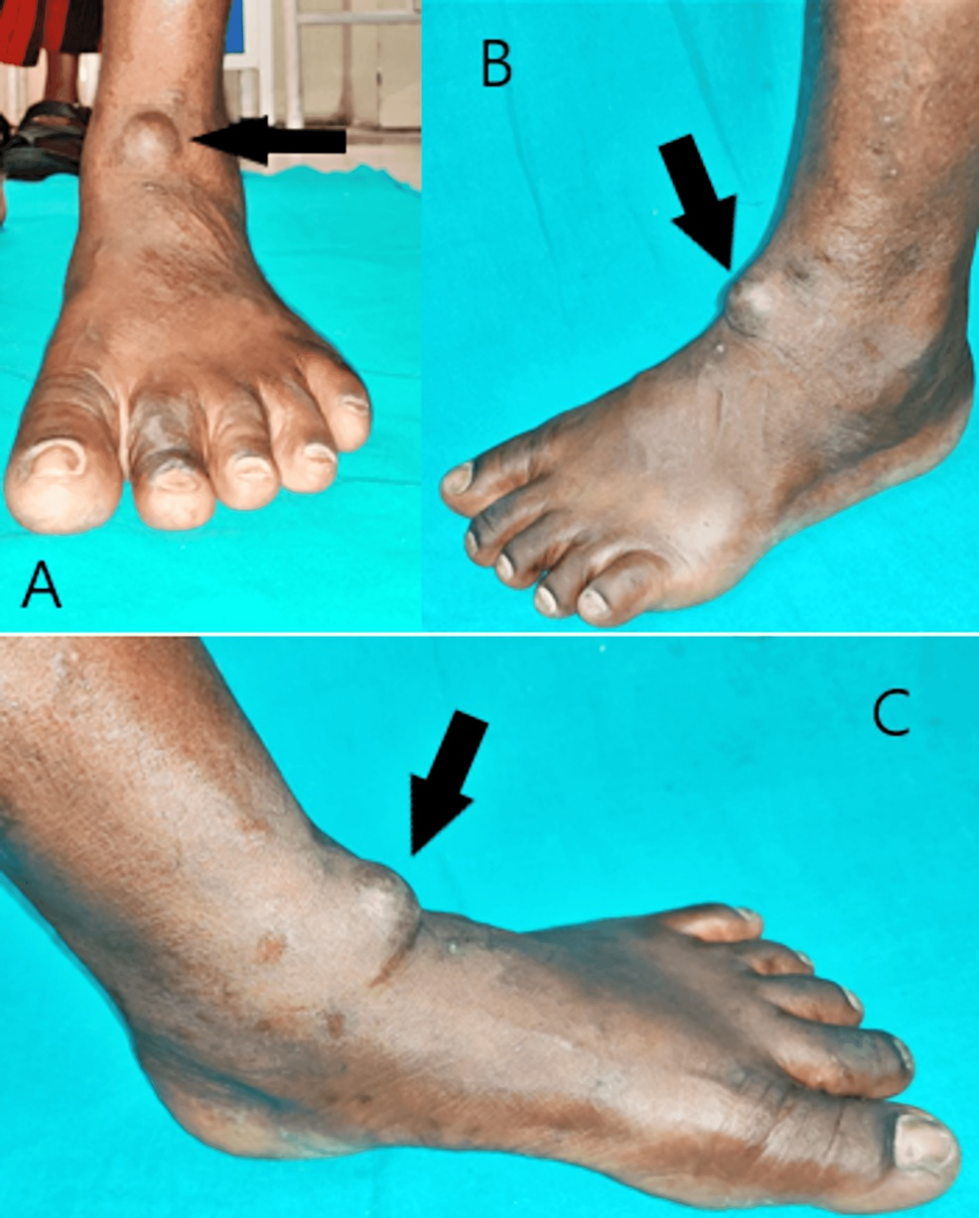
Clinical images of the tumor over the extensor aspect of the left ankle A: anterior aspect; B: lateral aspect; C: medial aspect

A 42-year-old male with no remarkable medical history visited the hospital with a painless mass on the extensor aspect of his left foot and slow growth for the past few years. Upon general examination, there was no abnormality detected. On local examination, there was a swelling of approximately 2 x 2 cm over the dorsal aspect of the left ankle (Figure 1).

The mass was well-defined and sharply demarcated. The overlying skin was not tense, and there was no local rise in temperature. It was soft, non-tender, non-pulsatile, mobile, and not adherent to the overlying skin or underlying deep fascia. The mass was ovoid in shape with clear, well-defined edges and a soft, doughy consistency. Fluctuation as well as the trans-illumination test were negative.

**Figure 2:**
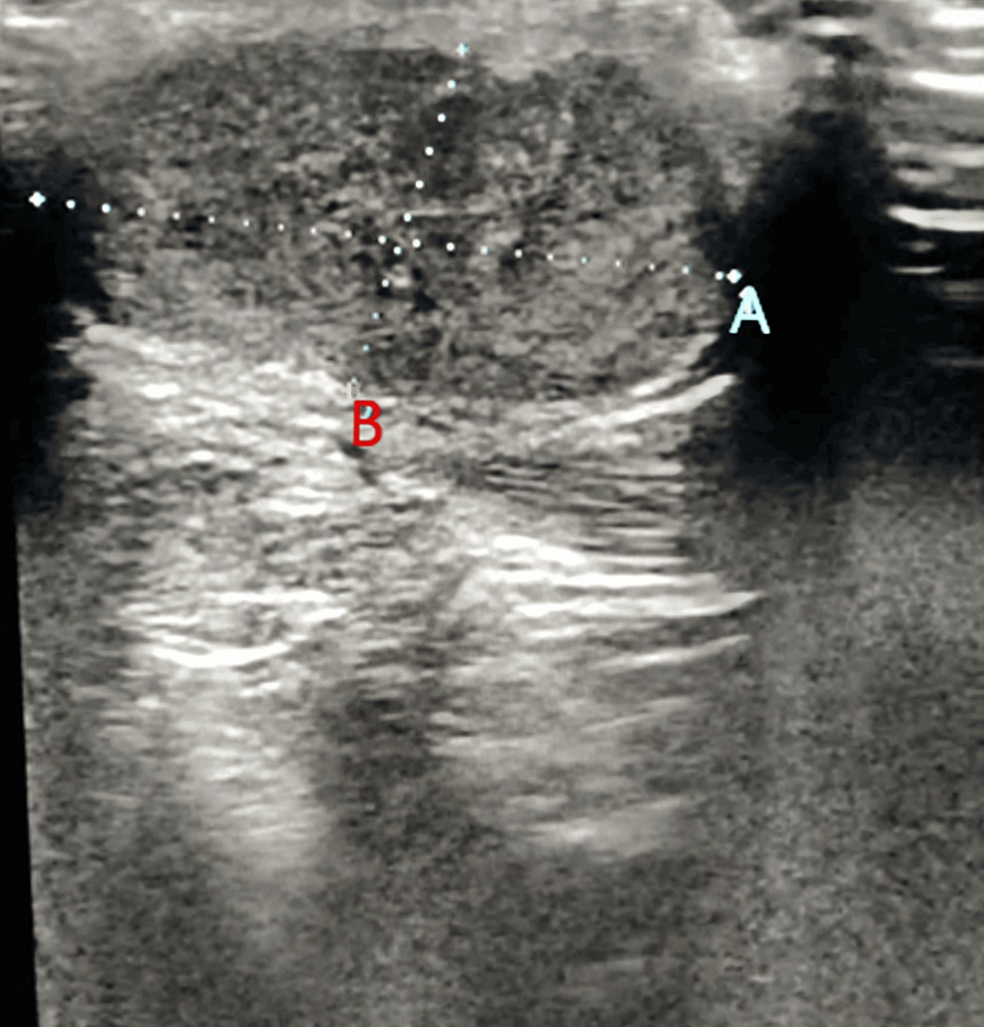
An ultrasound image of the tumor Panes A and B show the length and width of the lesion, respectively.

Ultrasound examination revealed a well-defined heterogeneously hypoechoic lesion of size 1.9 x 2.2 cm in the subcutaneous plane of the extensor aspect of the left ankle (Figure 2).

It was hard on the elastography. The differential diagnoses were lipoma, sebaceous cyst, ganglion cyst, and fibroma. A histopathological examination was advised for confirmation of the diagnosis.

**Figure 3:**
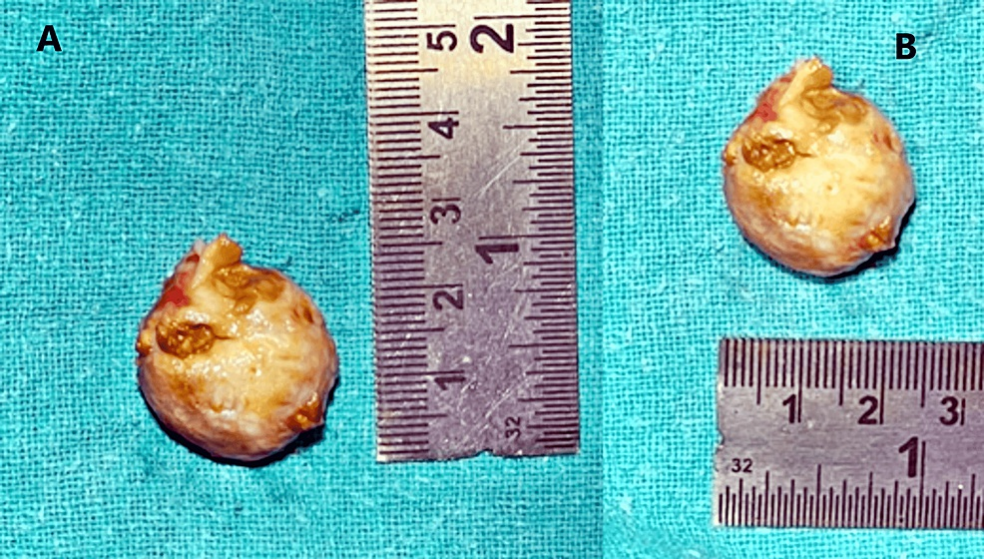
Panes A and B show the excised, encapsulated tumor of size 2.5 x 2.1 cm.

The patient was then scheduled for surgical excision of the tumor. Intra-operatively, an encapsulated mass of size 2.5 x 2.1 cm was removed from the subcutaneous plane over the extensor aspect of the left ankle (Figure 3).

**Figure 4:**
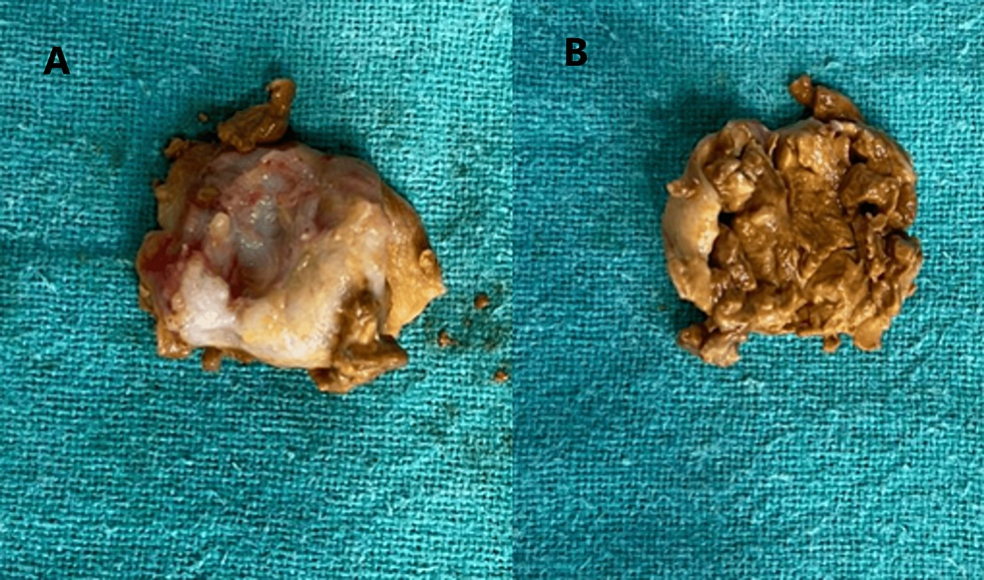
The cut section of the excised tumor with yellowish-brown tissues A: external aspect of the tumor; B: internal aspect of the tumor

The surrounding tissues were cauterized to prevent a recurrence. The excised tumor was then cut open, and it showed multiple nodules and pockets of yellowish-brown material (Figure 4).

The tumor excised was then sent for histopathological examination to confirm the diagnosis.

**Figure 5:**
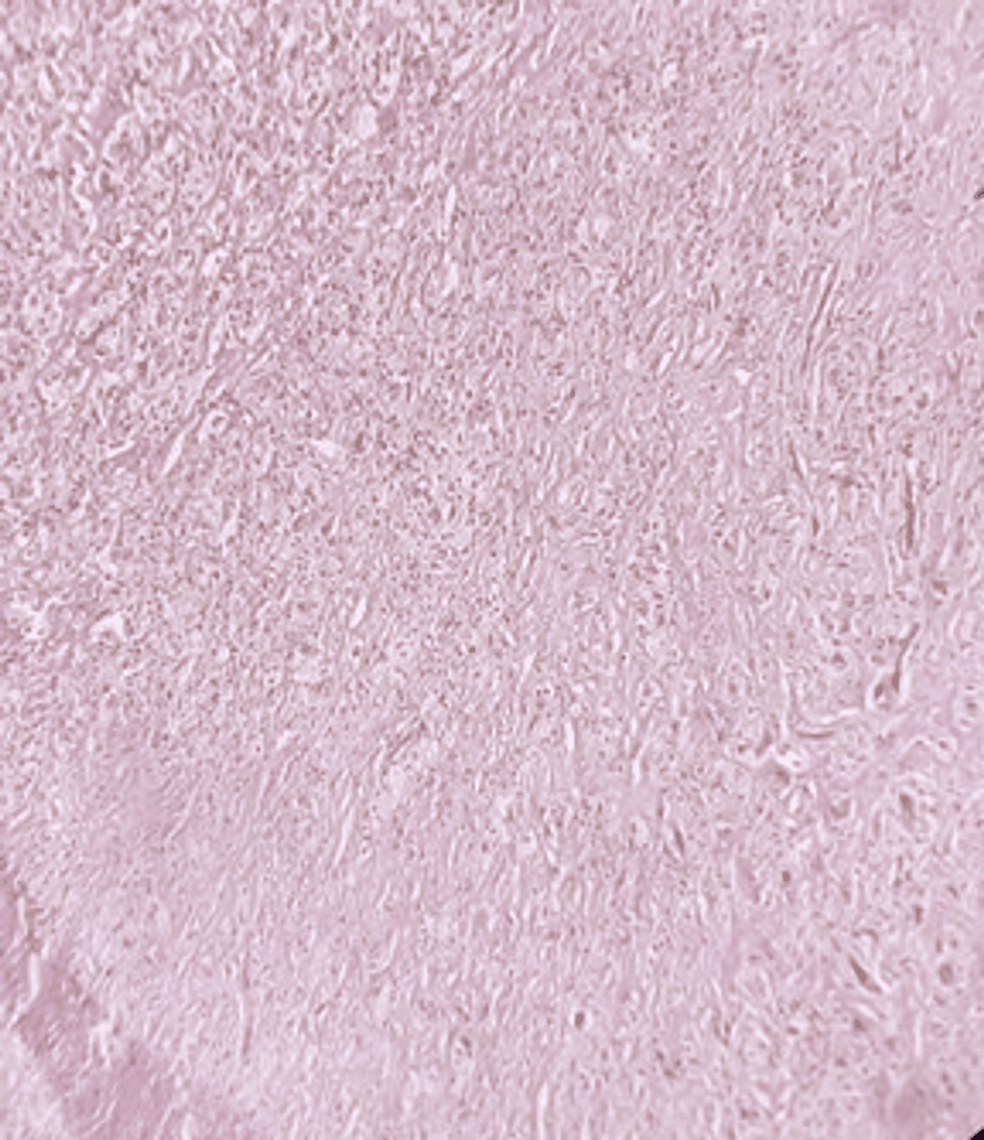
Histopathological image of the angiofibroma (H&E stained, 40X)

Histopathological examination revealed multiple lobules with well-circumscribed, alternating areas of collagenous and myxoid tissues. There was a prominent small vascular network with uniformly arranged spindle cells consisting of pale eosinophil-rich cytoplasm and small ovoid nuclei, fine chromatin, and an indistinct nucleolus (Figure 5).

Post-operatively, the patient was discharged on day 12 following suture removal. The patient was then followed up at the third week, two months, and six months. At his most recent one-year follow-up, there was a visible healed suture scar over the anterior aspect of the ankle. There was no evidence of swelling, recurrence, or infection. The patient was able to do his routine activities without any discomfort. All ankle range of movements were full and painless.

## Discussion

In a group of 37 instances, angiofibroma of soft tissue was first identified as a rare benign fibrous vascularized tumor in 2012 [1]. It manifests as deep-seated, slowly developing tumors in the extremities, primarily affecting the lower limbs [1]. The back, chest wall, abdominal wall, and pelvic cavity can also encounter it [3]. In the cases reported to date, there seems to be a female predominance, which is twice as often as males [4]. The majority of these tumors were well confined, with an average tumor size of 3.5 cm [1]. In our case, the tumor’s largest dimension was 2.5 cm. A somewhat homogeneous multiplication of homogenous spindle cells with unremarkable cytoplasm and an oblong-shaped nucleus embedded in a variable myxoid or collagenous stroma make up the lesion's two major microscopic features [2]. A distinct vascular architecture made up of a large number of tiny, branched, thin-walled vascular systems can be recognized [2]. These vessels are frequently joined by intermediate circular, asymmetrical, and ectatic veins. Rarely, infrequent mitosis (1-4/10 HPF) could be present [5]. Although it is unusual, a slight degenerative nuclear atypia might be found.

In our case, a confined tumor was evident, along with a proliferation of monotonous, uniformly shaped cells with barely discernible cytoplasm and oval nuclei. The stroma consisted of a mixed myxoid and collagenous matrix and had finely branching, thin-walled, tiny blood vessels. Mitoses were not encountered in the present case, and there were no signs of cellular atypia.

Angiofibroma of soft tissue can easily be confused with the following differential diagnoses: solitary fibrous tumor (SFT), myxoid liposarcoma, myxoid fibrosarcoma, and sebaceous cyst [1]. Hence, histopathological examination should be performed in suspected cases, and if needed, the positivity of CD34 foci should be checked in these tumors.

Management of these tumors consists of either a simple or wide excision. In cases with incomplete excision, the tumor might recur [6]. The patient described here had undergone a complete surgical excision with cauterization of the tumor bed and surrounding tissues to ensure no residual cells were left behind. At his most recent one-year follow-up, there was no evidence of recurrence.

## Conclusions

Angiofibroma is a benign soft tissue tumor consisting of fibrous vascular tissues that may recur locally in cases where partial or incomplete excision is done. There are not many reported cases of this clinical entity, and every new case reported brings light to the pathology and progression of this tumor. Understanding this pathology is necessary since it mimics many other skin and soft tissue tumors.
